# Real-world ADHD pharmacological treatment patterns and their association with negative clinical outcomes in youth with comorbid autism: a Swedish population-based study

**DOI:** 10.1136/bmjment-2026-302902

**Published:** 2026-08-11

**Authors:** Miguel Garcia-Argibay, Ralf Kuja-Halkola, Brian M D’Onofrio, Paul Lichtenstein, Zheng Chang, Henrik Larsson, Samuele Cortese

**Affiliations:** 1 Developmental EPI (Evidence synthesis, Prediction, Implementation) lab, Centre for Innovation in Mental Health, Faculty of Environmental and Life Sciences, University of Southampton, Southampton, UK; 2 Centre for Population Health, Research Department, Division for Mental Health, Haukeland University Hospital, Bergen, Norway; 3 School of Medical Sciences, Faculty of Medicine and Health, Örebro University, University, Sweden; 4 Department of Medical Epidemiology and Biostatistics, Karolinska Institutet, Stockholm, Sweden; 5 Department of Psychological and Brain Sciences, Indiana, Indiana University, Bloomington, Indiana, USA; 6 Hampshire and Isle of Wight NHS Foundation Trust, Southampton, UK; 7 Clinical and Experimental Sciences (CNS and Psychiatry), Faculty of Medicine, University of Southampton, Southampton, UK; 8 Hassenfeld Children’s Hospital at NYU Langone, New York University Child Study Center, New York City, NY, USA; 9 DiMePRe-J-Department of Precision and Regenerative Medicine-Jonic Area, University of Bari "Aldo Moro", Bari, Italy

**Keywords:** Attention Deficit and Disruptive Behavior Disorders, Psychopharmacology

## Abstract

**Background:**

Attention-deficit/hyperactivity disorder (ADHD) medications can reduce ADHD symptom severity in individuals with comorbid autism spectrum disorder (ASD). However, clinical guidance on pharmacological treatment of ADHD in this clinical population remains limited and inconsistent. Characterising real-world treatment patterns (ie, initiation timing, medication choices, switching, discontinuation) and the impact of alternative medication choices on clinical outcomes is critical for informing evidence-based management strategies.

**Objective:**

To (1) characterise ADHD pharmacological treatment patterns in youth with ADHD+ASD versus ADHD alone and (2) assess whether using alternative ADHD medications versus methylphenidate is associated with differential changes in negative clinical outcomes among youth with ADHD+ASD.

**Methods:**

This is a population-based cohort study using Swedish national registers. The study included children (<13 years) and adolescents (13–17 years) with an incident ADHD diagnosis between 2007 and 2018 and followed-up until 2021, comparing youth with co-occurring ASD (n=24 117) and ADHD alone (n=79 830). Descriptive outcomes included time to pharmacological treatment initiation, medication type, number of medication switches and discontinuations. The primary outcome was changes in rates of inpatient psychiatric hospitalisations, accidental injuries and specialist care visits for substance use, depressive or anxiety disorders in the 1 year after versus the 1 year before medication initiation.

**Findings:**

Individuals with ADHD+ASD experienced longer delays to treatment initiation (12–14% initiated >12 months after diagnosis vs 7–8% in ADHD alone). Children with ADHD+ASD were slightly more likely to discontinue treatment within 3 months (16% vs 12%) and had the highest average number of medication switches within 3 years (2.6; IQR 0.0–2.0). In within-individual analyses, comparisons of alternative ADHD medications versus methylphenidate did not yield statistically significant differences after correcting for multiple comparisons.

**Conclusions:**

Children with ADHD+ASD experienced longer delays to treatment initiation and more frequent medication switching compared with those with ADHD only. The effects of alternative ADHD medication options on key negative clinical outcomes appeared similar to those of methylphenidate.

**Clinical implications:**

These findings suggest that, rather than recommending fixed first-line and second-line treatments for individuals with ADHD-ASD, clinical guidelines should emphasise appropriate training as well as prompt and individualised treatment based on a shared decision-making process.

**WHAT IS ALREADY KNOWN ON THIS TOPIC**

- Attention-deficit/hyperactivity disorder (ADHD) frequently co-occurs with autism spectrum disorder (ASD), and ADHD medications reduce ADHD symptoms in autistic individuals, though with smaller effect sizes and poorer tolerability than in ADHD alone. Clinical guidelines specify a general first-line/second-line medication hierarchy for ADHD but provide no ASD-specific treatment algorithm; real-world evidence on treatment patterns and comparative outcomes in ADHD+ASD is limited, with prior work examining initiation but not switching, discontinuation or downstream clinical outcomes.

**WHAT THIS STUDY ADDS**

- In this population-based cohort study, youth with ADHD+ASD experienced longer delays to treatment initiation and more medication switches compared with those with ADHD alone. Use of alternative ADHD medications (eg, atomoxetine, lisdexamfetamine) was not associated with significant differences compared with methylphenidate in rates of psychiatric hospitalisations, injuries and specialist visits for depression.

**HOW THIS STUDY MIGHT AFFECT RESEARCH, PRACTICE OR POLICY**

- Pharmacological treatment patterns are more complex for youth with ADHD+ASD compared with ADHD alone. The effects of alternative ADHD medication options on key negative clinical outcomes appear largely similar to those of methylphenidate. On safety grounds, treatment selection in this population can be individualised through shared decision-making and supported by adequate clinician training; comparative efficacy, which underpins the rationale for first-line stimulant use, was not assessed here.

## Background

 Attention-deficit/hyperactivity disorder (ADHD) is the most prevalent neurodevelopmental condition and interferes with daily functioning and causes substantial impairment across multiple life domains.^1–4^ Pharmacological treatment is an important component of multimodal management, with stimulants generally recommended as first-line option and non-stimulants as second-line option in clinical guidelines.^5 6^

ADHD frequently co-occurs with other neuropsychiatric and physical conditions,^4 7^ with autism spectrum disorder (ASD) receiving growing attention. Meta-analytical evidence suggests that up to 21% of individuals with ADHD also meet criteria for ASD.^8^ This comorbidity has important implications for clinical practice and highlights the need to understand how comorbid ASD may influence the effects of ADHD treatment. However, current clinical guidance on ADHD treatment specifically in individuals with comorbid ASD is limited and inconsistent. For instance, the American Academy of Pediatrics guidelines^5^ report strong evidence for stimulants and less strong evidence for non-stimulants for children with ADHD generally but do not specifically address the management of children with comorbid ADHD-ASD. The Canadian ADHD Resource Alliance guidelines^9^ summarise key literature from randomised controlled studies but offer no specific guidance on treatment selection. The National Institute for Health and Care Excellence guidelines^10^ recommend a specific medication hierarchy for school-aged children with ADHD (methylphenidate first, followed by lisdexamfetamine/amphetamine and then non-stimulants). For cases with comorbid autism, the same options are suggested without an ASD-specific algorithm.

Regarding the evidence informing clinical recommendations, a meta-analysis of short-term randomised controlled trials (RCTs) indicated that stimulants and non-stimulants reduce ADHD symptom severity in individuals with ASD—although with an effect size generally lower than those found in children with ADHD only^6^—and were generally well tolerated, but generally less well than in children with ADHD only.^11^ Given that RCTs of ADHD medications are hampered by poor real-world representativeness,^12^ their results should be triangulated with those from observational studies. The latter provide insights into real-world outcomes and into treatment patterns, including initiation timing, medication choices, switching and discontinuation, thus providing key evidence to inform clinical management. One prior observational study^13^ found that individuals with ADHD-ASD were less likely to initiate methylphenidate and more likely to receive second-line treatments at lower doses. Critically, however, this study did not examine dynamic aspects of treatment such as medication switching or discontinuation, nor did it assess how different initial medication choices impact clinical outcomes. Consequently, key indicators of ADHD-related morbidity, including psychiatric crises, accidental injuries and treatment for co-occurring mental health disorders, remain unexplored.

Building on this knowledge gap, this study aimed to (1) characterise and compare pharmacological treatment patterns in youth with ADHD+ASD and in those with ADHD alone and (2) assess whether using alternative ADHD medications is associated with a significant change in rates of negative clinical outcomes compared with initiating methylphenidate in youth with ADHD+ASD using a within-individual design aimed to minimise confounders.

## Methods

### Study population

This study used data from linked Swedish national registers. The source population included all individuals residing in Sweden. We used the National Patient Register, the Prescribed Drug Register and the Total Population Register. Linkage between these registers was performed using the unique personal identification number assigned to all Swedish residents. Additional information on the registers can be found in the online supplemental material.

**Figure 1:**
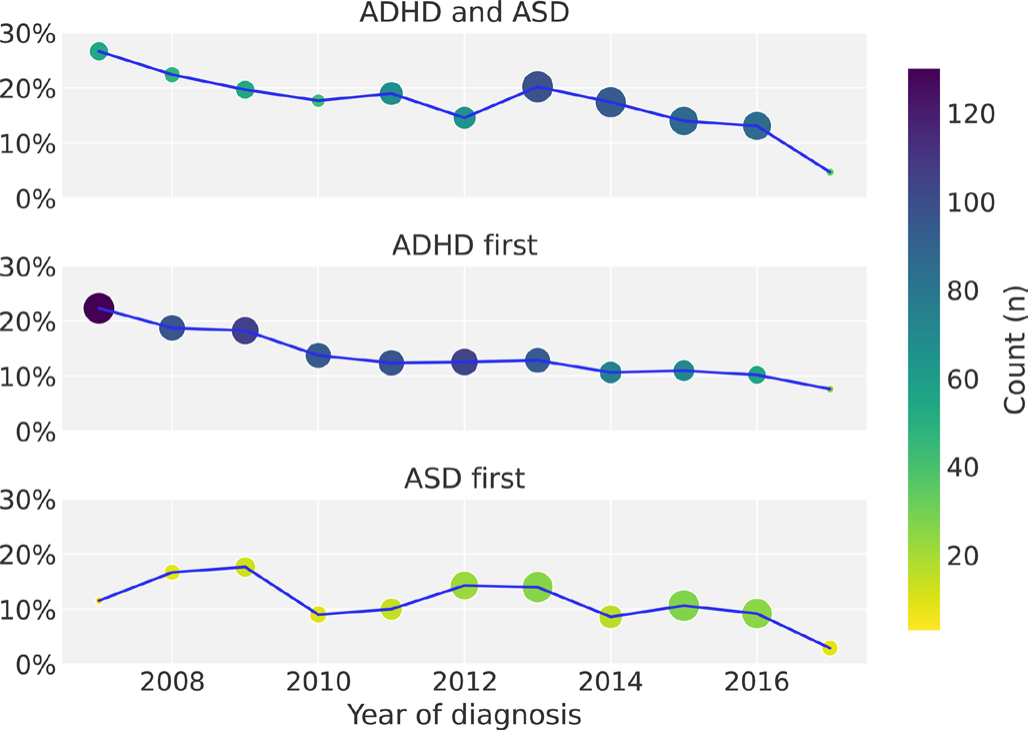
Proportion of individuals experiencing treatment delay longer than 12 months from ADHD diagnosis stratified by order of diagnosis. ADHD, attention-deficit/hyperactivity disorder; ASD, autism spectrum disorder.

The study cohort included children and adolescents (aged <18 years) with an ADHD diagnosis (International Classification of Diseases 10th Revision (ICD-10) code F90) between 1 January 2007 and 31 December 2018, with follow-up until 2021. Individuals were further classified based on the presence or absence of a co-occurring ASD (F84) diagnosis. The index date was defined as the date of the first recorded ADHD diagnosis. For the ADHD+ASD group, the diagnostic sequence (ADHD first, ASD first or concurrent) was also determined. The cohort was stratified into children (age <13 years at index) and adolescents (ages 13–17 years at index) for descriptive analyses. Because ASD may be recorded before, concurrently with or after the ADHD diagnosis, ASD status was defined over the entire observation period and may not have been clinically recognised at the ADHD index date; we therefore characterised the diagnostic sequence (ASD first, concurrent, ADHD first) and examined delayed initiation separately by sequence (figure 1). An overview of the study design and the different index dates used for each aim is provided in online supplemental figure 1.

### Exposure and outcomes

Exposure to ADHD medication included stimulants (methylphenidate, dexmethylphenidate, amphetamine, dexamphetamine and lisdexamfetamine) and non-stimulants (atomoxetine and guanfacine). For each dispensed prescription, the date of dispensation and the total number of defined daily doses dispensed were used to estimate the duration of supply.

Treatment initiation was identified as the first dispensed prescription for any ADHD medication following the index ADHD diagnosis. The time-to-treatment initiation was calculated as the interval from the index ADHD diagnosis to the first ADHD medication dispensation date and was categorised for descriptive analysis as occurring within the first 3 months, between 4 and 12 months or after more than 12 months. For the ADHD+ASD group specifically, temporal trends in delayed initiation (defined as >12 months) were further examined according to the year of ADHD diagnosis and the diagnostic sequence (ADHD first, ASD first or concurrent).

Changes in medication regimens were captured by defining a medication switch as the initiation of different medication within the same class or a different class after a period of use of a prior ADHD medication. The number of such switches occurring within the first 3 years after index ADHD diagnosis was then calculated for each individual. A 3-year follow-up period after index ADHD diagnosis was chosen for quantifying medication switches and discontinuations to provide a comprehensive picture of treatment dynamics beyond the initial titration and adjustment phase. This duration enabled us to capture immediate treatment responses and early discontinuations and to capture longer term patterns of medication sequencing, persistence and potential reinitiation after a period off treatment, offering a more robust view of an individual’s medication trajectory. As a sensitivity analysis addressing differential time at risk for switching, the number of switches was also expressed as a rate, with the at-risk time from the first medication dispensation to the end of the 3-year window included as an offset (quasi-Poisson regression with family-clustered SEs; online supplemental table 2).

Treatment discontinuation was defined as a gap exceeding 90 days between the expected end date of a dispensed prescription and the date of the next dispensed prescription for any ADHD medication, in line with previous studies.^12 14^ The expected end date of a given prescription was determined by adding its estimated duration of supply (the number of days the dispensed medication was intended to last) to the date of its collection from the pharmacy. The number of these discontinuation episodes within the first 3 years following the index ADHD diagnosis was also quantified.

Negative clinical outcomes were included: inpatient psychiatric hospitalisations, defined by any primary or secondary discharge diagnosis; emergency department visits or hospitalisations related to accidental injuries or accidents; and specialist care visits with a primary or secondary diagnosis of alcohol-related disorders, drug-related disorders excluding alcohol and caffeine, depressive disorders including dysthymia and anxiety disorders including phobic anxiety disorders and obsessive-compulsive disorder. Notably, there are established mechanistic links between ADHD medication and the risk for these events.^15^ The frequency of these outcomes in our population-based cohort ensured sufficient statistical power for meaningful analysis, unlike rarer comorbidities.

For each individual initiating ADHD medication, all defined clinical outcomes were assessed and counted separately during the 1-year period immediately before treatment initiation and the 1-year period immediately after treatment initiation.^12^ The first dispensation date served as the anchor point. Follow-up ended at emigration, death, treatment discontinuation or 1 year after initiation, whichever occurred first. Additional details on the rationale for our methodological choices are provided in the online supplemental methods, and ICD-10 codes used are listed in online supplemental table 1.

### Statistical analysis

Baseline characteristics were summarised by diagnostic (ADHD+ASD or ADHD alone) and age (children <13 years; adolescents 13–17 years) group. Frequencies and percentages were reported for categorical variables. The distribution of pharmacological agents used was described for first-line treatment as well as for second-line medication choices among individuals who subsequently switched therapies. For continuous or count variables, such as age at index, the number of medication switches or discontinuations within 3 years, means and IQRs were presented.

To compare the impact of initiating different ADHD medications relative to methylphenidate (the most common first-line treatment), we employed a within-individual (self-controlled) design restricted to each individual’s first-ever ADHD medication, in which each individual serves as their own control, so all time-invariant individual-level characteristics (eg, sex, genetic background and stable family and socioeconomic factors) are accounted for by design. We compared the rate of each adverse outcome during the 1-year period immediately before medication initiation (reference period) to the rate in the 1-year period immediately after. This was modelled using quasi-Poisson regression with log link, with incidence rate ratios (IRRs) and 95% CIs derived from an interaction term between medication type and the time-period indicator (preinitiation vs postinitiation). Overdispersion in the outcome count data was addressed using a quasi-Poisson scale parameter. To account for differences in individual follow-up durations due to censoring (emigration, death, treatment discontinuation), the natural logarithm of person-time was included as an offset. Finally, to account for non-independence of observations within families, we used two-way cluster-robust (sandwich) SEs, with individual and family units defined as clusters. To control for the false discovery rate given the multiple comparisons performed, we applied the Benjamini-Hochberg (BH) procedure. Analyses were performed using R V.4.4.3.^16^

In the within-individual analysis (aim 2), restricted to youth with ADHD+ASD who initiated pharmacotherapy with their first-ever ADHD medication, the index date was the first medication dispensation rather than the ADHD diagnosis, because the question concerns the consequences of the initial medication choice; adverse outcome rates were compared in the year before versus the year after initiation.

## Findings

### Treatment patterns

The study cohort comprised 103 947 individuals, including 14 654 children (<13 years) and 9463 adolescents (aged 13–17 years) with co-occurring ADHD and ASD, and 37 972 children and 41 858 adolescents with ADHD alone. Males were predominant across all groups, although the proportion was lower among adolescents than children in both the ADHD+ASD group (60% vs 78%) and the ADHD-only group (57% vs 75%). The mean age (IQR) at ADHD diagnosis was 8.6 (7.1–10.2) years for children and was 14.8 (13.4–16.2) years for adolescents with ADHD+ASD; for the ADHD-alone group, mean ages were 9.2 (7.9–10.7) years for children and 15.0 (13.6–16.4) years for adolescents.

Within the ADHD+ASD group, the temporal sequence of diagnosis varied slightly by age: concurrent ADHD and ASD diagnosis was reported for 37% of children and 39% of adolescents, while an ADHD diagnosis preceded ASD for 44% of children and 37% of adolescents. An ASD diagnosis first was reported for 19% of children and 24% of adolescents with ADHD+ASD (table 1).

**Table 1:** Descriptive statistics of the study cohort stratified by the presence of comorbid ASD for children and adolescents with ADHD

Characteristic	Comorbid ADHD+ASD	ADHD alone	P value	Comorbid ADHD+ASD	ADHD alone	P value
	Children	Children		Adolescents	Adolescents	
	n=14 654	n=37 972		n=9463	n=41 858	
Sex			<0.001			<0.001
Male	11 392 (78%)	28 493 (75%)		5725 (60%)	23 694 (57%)	
Female	3262 (22%)	9479 (25%)		3738 (40%)	18 164 (43%)	
Age, years	8.6 (7.1, 10.2)	9.2 (7.9, 10.7)	<0.001	14.8 (13.4, 16.2)	15.0 (13.6, 16.4)	<0.001
Diagnosis order						
ADHD and ASD	5433 (37%)	–		3715 (39%)	–	
ADHD first	6375 (44%)	–		3474 (37%)	–	
ASD first	2846 (19%)	–		2274 (24%)	–	
Started medication	12 428 (85%)	32 009 (84%)	0.15	7881 (83%)	36 442 (87%)	<0.001
Number of switches	2.6 (0.0, 2.0)	2.1 (0.0, 2.0)	<0.001	1.1 (0.0, 1.0)	1.0 (0.0, 1.0)	0.8
Number of discontinuations	0.56 (0.00, 1.00)	0.56 (0.00, 1.00)	0.069	0.58 (0.00, 1.00)	0.64 (0.00, 1.00)	<0.001
Time to treatment*			<0.001			<0.001
First 3 months	6055 (70%)	19 992 (77%)		3844 (70%)	23 070 (80%)	
4–12 months	1501 (17%)	3828 (15%)		935 (17%)	3650 (13%)	
>12 months	1077 (12%)	1989 (7.7%)		748 (14%)	2079 (7.2%)	
Initial medication			<0.001†			<0.001†
Amphetamine	<5 (<0.1%)	<5 (<0.1%)		<5 (<0.1%)	<5 (<0.1%)	
Atomoxetine	1092 (8.8%)	1972 (6.2%)		752 (9.5%)	2513 (6.9%)	
Dexmethylphenidate	<5 (<0.1%)	5 (<0.1%)		<5 (<0.1%)	6 (<0.1%)	
Guanfacine	68 (0.5%)	121 (0.4%)		38 (0.5%)	94 (0.3%)	
Lisdexamfetamine	156 (1.3%)	471 (1.5%)		175 (2.2%)	922 (2.5%)	
Methylphenidate	11 107 (89%)	29 439 (92%)		6912 (88%)	32 906 (90%)	
Second medication‡			<0.001§			<0.001§
Amphetamine	7 (<0.1%)	5 (<0.1%)		<5 (0.1%)	9 (<0.1%)	
Atomoxetine	3873 (50%)	6833 (40%)		1404 (38%)	5594 (35%)	
Dexmethylphenidate	34 (0.4%)	102 (0.6%)		32 (0.9%)	106 (0.7%)	
Guanfacine	378 (4.9%)	715 (4.2%)		93 (2.5%)	316 (2.0%)	
Lisdexamfetamine	2654 (34%)	7941 (46%)		1635 (44%)	8128 (51%)	
Methylphenidate	835 (11%)	1555 (9.1%)		510 (14%)	1917 (12%)	
Time to discontinuation			<0.001			<0.001
First 3 months	1948 (16%)	3753 (12%)		1240 (16%)	5507 (15%)	
4–12 months	1618 (13%)	4380 (14%)		1355 (17%)	7095 (19%)	
>12 months	3145 (25%)	7655 (24%)		1599 (20%)	6856 (19%)	
Continued	5717 (46%)	16 221 (51%)		3686 (47%)	16 982 (47%)	

Values are presented as n (%) for categorical variables and mean (IQR) for continuous variables (age, number of switches, number of discontinuations).

* Estimated among those with an ADHD diagnosis prior starting medication.

† Excluding amphetamine and dexmethylphenidate.

‡ Among individuals who switched from their first ADHD medication.

§ Excluding amphetamine.

ADHD, attention-deficit/hyperactivity disorder; ASD, autism spectrum disorder.

In terms of pharmacological treatment initiation, a notable delay was observed in the ADHD+ASD cohort compared with the ADHD-alone cohort. Time to treatment could be calculated only for individuals whose recorded ADHD diagnosis preceded their first ADHD medication dispensation (children with ADHD+ASD: 8633 of 12 428 treated; adolescents with ADHD+ASD: 5527 of 7881; children with ADHD alone: 25 809 of 32 009; adolescents with ADHD alone: 28 799 of 36 442); the remainder had a first dispensation recorded before their first specialist-registered ADHD diagnosis—most likely reflecting diagnosis in primary care or treatment initiated during the diagnostic work-up before the specialist code was entered—and were excluded from this calculation. Among these individuals, 70% of children with ADHD+ASD (6055/8633) initiated within 3 months, compared with 77% of children with ADHD alone (19 992/25 809); the same pattern was seen in adolescents (ADHD+ASD 70%, 3844/5527; ADHD alone 80%, 23 070/28 799). Accordingly, a larger proportion of the ADHD+ASD group experienced longer delays; 12% of children and 14% of adolescents with ADHD+ASD initiated treatment after more than 12 months, compared with 7.7% of children and 7.2% of adolescents in the ADHD-alone group. Further examination of this delayed initiation (>12 months) within the ADHD+ASD group by year of diagnosis and diagnostic temporal sequence revealed distinct temporal trends (figure 1). For individuals diagnosed concurrently with ADHD and ASD, the percentage experiencing a >12 months’ treatment delay generally decreased over time, from 27% for those diagnosed in 2007 to 5% by 2016. A similar, but less steep, declining trend in delayed initiation was observed for those diagnosed with ADHD first (22% to 8%). In contrast, for individuals diagnosed with ASD first, the percentage experiencing a >12 months’ delay in subsequent ADHD treatment initiation showed more fluctuation, with peaks around 18% in 2009 and 14% in 2012–2013, before also declining in later years.

Among those treated, methylphenidate was the most common initial prescription across all subgroups, ranging from 88% in adolescents with ADHD+ASD to 92% in children with ADHD alone (table 1). Atomoxetine was more frequently prescribed as a first-line agent in the ADHD+ASD cohort (8.8% for children, 9.5% for adolescents) compared with the ADHD-alone cohort (6.2% for children, 6.9% for adolescents), although the difference between the two groups was small. When examining subsequent medication options for the ADHD+ASD group among those who switched medications, patterns varied by age. Among children with ADHD+ASD switching to another medication, atomoxetine was the most common (50%), followed by lisdexamfetamine (34%). For adolescents with ADHD+ASD, lisdexamfetamine was more prominent as a second choice (44%), followed by atomoxetine (38%). Similar patterns of methylphenidate dominance first-line treatment, followed by atomoxetine and lisdexamfetamine in subsequent options, were observed in the ADHD-alone group, although with some age-related variations in preference for second-line agents (eg, higher lisdexamfetamine use as a second medication in ADHD-alone adolescents (51%) compared with ADHD-alone children (46%)).

The mean number of medication switches within 3 years was higher in children with ADHD+ASD (2.6, IQR 0.0–2.0), compared with adolescents with ADHD+ASD (1.1, IQR 0.0–1.0), children with ADHD alone (2.09, IQR 0.0–2.0) and adolescents with ADHD alone (1.03, IQR 0.0–1.0). Regarding treatment discontinuation patterns, children with ADHD+ASD were less likely to continue their initial treatment without discontinuation (46%) compared with children with ADHD alone (51%), adolescents with ADHD+ASD (47%) and adolescents with ADHD alone (47%). This pattern persisted when switching was expressed as a rate per person-year on treatment (children with ADHD+ASD vs ADHD alone: rate ratio 1.12, 95% CI 1.07 to 1.18; adolescents: 1.02, 95% CI 0.95 to 1.10; online supplemental table 2), confirming that it is not an artefact of differential time at risk. Furthermore, a higher proportion of children with ADHD+ASD discontinued treatment within the first 3 months (16%) compared with children with ADHD alone (12%) and adolescents with ADHD alone (15%). Discontinuation after more than 12 months of treatment was more common among children with ADHD+ASD (25%) and children with ADHD alone (24%) compared with their adolescent counterparts (ADHD+ASD adolescents: 20%; ADHD-alone adolescents: 19%). Discontinuation between 4 and 12 months showed less pronounced variation, with percentages ranging from 13% (children with ADHD+ASD) to 19% (adolescents with ADHD alone).

### Changes in rates of negative clinical outcomes

When examining how the change in outcome rates varied by specific medication type relative to methylphenidate, no associations retained statistical significance after applying the BH correction for multiple comparisons (table 2). In the uncorrected analysis, a few trends were observed. Initiation of guanfacine was nominally associated with a lower rate of psychiatric hospitalisation than methylphenidate, both overall (IRR=0.33, 95% CI 0.11 to 0.95) and among children (IRR=0.14, 95% CI 0.03 to 0.65); these estimates were based on few guanfacine initiators, did not survive the BH correction and should be regarded as hypothesis generating. Among adolescents with ADHD+ASD, the initiation of atomoxetine appeared to show a larger reduction in the rate of anxiety-related visits compared with methylphenidate (IRR=0.83, 95% CI 0.73 to 0.95, uncorrected p=0.007, p_BH_=0.10). Similarly, among children with ADHD+ASD, lisdexamfetamine was nominally associated with a less favourable change in depression-related visits compared with methylphenidate (IRR=1.79, 95% CI 1.22 to 2.61, uncorrected p=0.01, p_BH_=0.10).

**Table 2:** Results from the within-individual analysis displaying IRRs of clinical outcomes pretreatment versus post-treatment among individuals with comorbid ADHD and ASD, comparing each medication to methylphenidate

Outcome	Medication	Age		
		Overall	Children	Adolescents
Accidents/injuries	Atomoxetine	0.95 (0.75 to 1.21)	1.11 (0.80 to 1.52)	0.78 (0.56 to 1.08)
Accidents/injuries	Guanfacine	0.94 (0.41 to 2.14)	0.89 (0.32 to 2.48)	0.97 (0.24 to 3.89)
Accidents/injuries	Lisdexamfetamine	1.08 (0.69 to 1.69)	0.62 (0.30 to 1.27)	1.37 (0.74 to 2.51)
Anxiety	Atomoxetine	0.85 (0.75 to 0.95)	0.84 (0.67 to 1.05)	0.83 (0.73 to 0.95)
Anxiety	Guanfacine	0.99 (0.74 to 1.32)	0.98 (0.56 to 1.73)	0.99 (0.71 to 1.37)
Anxiety	Lisdexamfetamine	1.08 (0.89 to 1.30)	1.13 (0.74 to 1.71)	1.05 (0.85 to 1.29)
Depression	Atomoxetine	0.87 (0.75 to 1.01)	0.85 (0.57 to 1.27)	0.87 (0.75 to 1.01)
Depression	Guanfacine	0.99 (0.64 to 1.54)	1.20 (0.43 to 3.36)	0.99 (0.62 to 1.58)
Depression	Lisdexamfetamine	1.04 (0.87 to 1.24)	1.79 (1.22 to 2.61)	1.02 (0.86 to 1.22)
Inpatient	Atomoxetine	0.67 (0.44 to 1.01)	0.59 (0.26 to 1.32)	0.72 (0.46 to 1.15)
Inpatient	Guanfacine	0.33 (0.11 to 0.95)	0.14 (0.03 to 0.65)	0.55 (0.14 to 2.13)
Inpatient	Lisdexamfetamine	1.45 (0.81 to 2.60)	3.47 (0.27 to 44.44)	1.45 (0.80 to 2.63)
SUD	Atomoxetine	0.6 (0.29 to 1.25)	NA	0.59 (0.29 to 1.23)
SUD	Guanfacine	1.18 (0.07 to 19.16)	NA	1.16 (0.07 to 18.87)
SUD	Lisdexamfetamine	0.88 (0.33 to 2.38)	NA	0.87 (0.32 to 2.35)

Coefficients are presented as IRR (95% CI).

NA denotes no convergence.

ADHD, attention-deficit/hyperactivity disorder; ASD, autism spectrum disorder; IRR, incidence rate ratio; SUD, substance use disorder.

## Discussion

This study was the first to investigate pharmacological treatment patterns in Swedish children and adolescents with ADHD with and without co-occurring ASD and to examine within-individual changes in real-world negative clinical outcomes related to different ADHD medication choices in those with ADHD+ASD.

Our findings suggest that, in Sweden and during the study period, clinicians largely follow the standard ADHD treatment hierarchy for this comorbid population, with modest adjustments towards non-stimulants like atomoxetine in a notable subset of cases. This result is overall in line with a similar pattern observed by Johansson *et al* in a Swedish cohort.^13^ However, we found no signal that initiating an alternative agent rather than methylphenidate was associated with a differential change in these adverse outcomes. Because our analysis addressed safety-related outcomes rather than ADHD symptom efficacy—on which the standard first-line preference for stimulants is principally based—these findings argue against avoiding alternative agents on safety grounds alone, rather than against the existence of a treatment hierarchy. Rather, an approach based on shared decision-making, taking into account individual patient preferences regarding the type of medication and the specific side effects to be avoided, would be preferable.

A key descriptive finding was the longer delay in treatment initiation observed among individuals with ADHD+ASD compared with those with ADHD alone. This delay may reflect parental or clinician caution, or challenges in accessing appropriate services for this dually diagnosed population. Of note, we found a generally decreasing trend in such delays over time, particularly for those diagnosed concurrently with ADHD and ASD or with ADHD first. This trend may reflect evolving clinical practice, increased awareness and improved pathways to care for ADHD in the context of ASD. A major contributing factor may have been the publication of Diagnostic and Statistical Manual of Mental Disorders, 5th Edition, in 2013,^17^ which removed the veto to codiagnose ADHD in individuals with ASD. This formally recognised co-occurrence of ADHD and ASD and contributed to facilitate earlier identification and treatment of ADHD symptoms in this population. Our results highlight the importance of continuing to raise awareness of the co-occurrence of ADHD and ASD (while at the same time acknowledging the issue of differential diagnosis) and providing training in their clinical management.

The mean number of medication switches within the first 3 years was slightly higher in the ADHD+ASD group than in the ADHD-alone group, particularly among children. This may indicate a more complex or challenging treatment optimisation process for individuals with dual diagnoses. This could reflect a greater need to trial different agents to achieve desired efficacy and/or manage tolerability issues specific to the co-occurrence of ADHD and ASD symptoms.^11^ These findings lend support to a treatment selection process based on shared decision-making, which has been found effective in decreasing treatment switches and discontinuation. For instance, in other fields of medicine, shared decision-making interventions can have a positive effect on patient adherence to disease-modifying drug therapy in multiple sclerosis care^18^ or adherence to pharmacotherapy in asthma treatment.^19^

Although the mean number of switches was statistically higher in children with ADHD+ASD, the median was 0 and the IQRs were identical to those of children with ADHD alone, indicating that this difference was driven by a minority of frequent switchers rather than a general shift. Given the large sample, this statistically significant difference is of modest clinical magnitude.

### Limitations

Some limitations should be acknowledged when interpreting the results. First, dispensed prescription data lack information on actual medication adherence, potentially affecting both treatment duration estimates and outcome associations. Second, our register data did not capture clinical reasons for medication initiation, switches or discontinuations (eg, inefficacy, adverse effects, patient preference, symptom resolution). Understanding these factors would provide richer context for observed medication patterns. Third, our ascertainment of outcomes such as depression and anxiety was based on specialist care visits. This approach captures clinically significant cases requiring specialist intervention but may not detect milder or subclinical symptoms, nor the fluctuating nature of these internalising disorders that do not result in a formal healthcare visit. Consequently, our findings on these outcomes are most relevant to more severe presentations. Fourth, infrequent first-line use of amphetamine and dexmethylphenidate in Sweden prevented their inclusion as comparators, limiting generalisability to settings where these are common alternatives. A further consideration is that, although the within-individual design removes confounding by all time-invariant individual characteristics, the comparison between medications is not randomised: the choice of an alternative agent over methylphenidate reflects clinical indication, symptom severity, tolerability and prior treatment response. Because the contrast is the difference between drug groups in their within-individual pre-to-post change, it assumes that, absent any true differential drug effect, the groups would have experienced parallel peri-initiation changes; severity-dependent regression to the mean and other time-varying, indication-related factors could violate this assumption, so residual confounding by indication cannot be excluded and the IRRs should not be interpreted as causal effects. Relatedly, the predominantly null findings should not be read uniformly as evidence of equivalence, nor uniformly as insufficient statistical power. For the most frequently used comparison (atomoxetine vs methylphenidate), CIs were comparatively narrow and excluded large differential effects, suggesting any true differential effect is likely modest. By contrast, most of the least used comparisons—the remaining guanfacine comparisons, all substance use outcomes (which did not converge among children) and lisdexamfetamine for psychiatric hospitalisation—had wide CIs, and these data are best regarded as inconclusive rather than as indicating similarity. The exception was the guanfacine–psychiatric hospitalisation association, which was nominally protective but rested on very few initiators and did not survive correction. Finally, this study did not measure treatment effectiveness regarding ADHD symptom reduction or functional improvements (eg, core symptoms, social functioning, attention). While observed changes in adverse clinical outcomes provide important perspective on medication impact, they do not offer a complete picture of overall treatment benefit or harms.

Because comorbid ASD was ascertained across the full observation period, it was not necessarily recognised clinically at the time of the ADHD diagnosis, particularly in the ADHD-first subgroup; treatment delays in that subgroup should therefore not be interpreted as a consequence of recognised comorbid ASD. Stratification of delayed initiation by diagnostic sequence (figure 1) partly mitigates this. Switches and discontinuations were counted within 3 years of the ADHD index date rather than from medication initiation; individuals who initiated later therefore had less time at risk for switching within this window, and some treatment decisions preceded the recording of ASD. These descriptive counts should be interpreted accordingly.

Finally, the observation period extended to 2021 and therefore overlapped with the COVID-19 pandemic. Pandemic-related disruptions to healthcare utilisation, including changes in diagnostic activity, specialist visits and medication dispensing, may have influenced the ascertainment of outcomes and observed treatment patterns towards the end of follow-up; however, because the data are population wide and both comparison groups were subject to the same secular conditions, this is unlikely to have introduced substantial differential bias between groups.

The within-individual comparison of initial medication choices was chosen to eliminate time-invariant unmeasured confounding by indication, which is particularly severe when comparing first-line medication choices in the clinically heterogeneous ADHD+ASD subgroup. Furthermore, a head-to-head target trial emulation with clone-censor-weighting in these small drug-specific subgroups would be substantially underpowered. Follow-up was censored at discontinuation and person-time was modelled as an offset, so the postinitiation period reflects time on the initial agent. Nonetheless, this design remains susceptible to time-varying confounding (eg, regression to the mean). This analysis is therefore exploratory and hypothesis generating, and confirmatory comparative safety and effectiveness studies (eg, target trial emulation) in larger samples are warranted.

## Conclusions

This study demonstrated that children with ADHD and comorbid ASD experienced longer delays to treatment initiation and more frequent medication switching compared with those with ADHD alone. Among youth with ADHD+ASD, initiating an alternative ADHD medication rather than methylphenidate was not associated with a differential change in psychiatric hospitalisation, accidental injuries or specialist visits for substance use, depressive or anxiety disorders, although precision was limited for the least used agents and for substance use outcomes. Combined with the absence of an ASD-specific algorithm in current guidelines, these findings suggest that, on safety grounds, treatment selection in this population can be individualised through shared decision-making and supported by adequate clinician training—while recognising that comparative efficacy, which underpins the rationale for first-line stimulant use, was not assessed here.

## Supplementary material

10.1136/bmjment-2026-302902 online supplemental file 1

## Data Availability

Data may be obtained from a third party and are not publicly available.
