# Supplementary material for: Real-world ADHD pharmacological treatment patterns and their association with negative clinical outcomes in youth with comorbid autism: a Swedish population-based study
Source: BMJ Ment Health. 2026 Aug 11;29(1):e302902. doi: 10.1136/bmjment-2026-302902 (PMC13475623; doi:10.1136/bmjment-2026-302902)
Supplement: Supplementary file 1 [file bmjment-29-1-s001.pdf]

Supplementary material for: **Real-world ADHD pharmacological treatment patterns and their association with negative clinical outcomes in youth with comorbid autism: a Swedish population-based study**

Miguel Garcia-Argibay et al. (2026)

Table of Contents

Supplementary Methods 2

Supplementary Table 1. Psychiatric diagnoses and negative outcomes by ICD-10 code 5

Supplementary Table 2. Medication switching expressed as a rate per person-year on treatment, by age and diagnostic group. 6

Supplementary Methods

This study utilized data from linked Swedish national registers. The source population included all individuals residing in Sweden. Individuals were identified through the National Patient Register (NPR), which contains information on all inpatient hospitalizations since 1987 and specialized outpatient clinic visits since 2001, including diagnostic codes according to the International Classification of Diseases (ICD). The Swedish Prescribed Drug Register (PDR), established in July 2005, provided data on all dispensed medications from pharmacies in Sweden, including Anatomical Therapeutic Chemical (ATC) classification codes, dispensation dates, and prescribed quantities. The Total Population Register provided demographic information, including date of birth and sex. Linkage between these registers was performed using the unique personal identification number assigned to all Swedish residents.

The selection of these specific clinical outcomes was a deliberate, a priori decision based on three core principles: high prevalence in the study population, established mechanistic links to ADHD and its treatment, and sufficient statistical power to detect meaningful change. First, the chosen outcomes represent the most common and clinically significant domains of morbidity and functional impairment associated with ADHD and co-occurring ASD. Psychiatric hospitalization serves as a robust, non-specific indicator of severe mental health crises, capturing the most acute events across all diagnostic categories. Accidental injuries are a well-documented consequence of the core ADHD symptoms of inattention and impulsivity, making their reduction a key goal of effective treatment. Finally, anxiety, depression, and substance use disorders are the most prevalent psychiatric comorbidities accompanying ADHD. Their inclusion is critical for understanding the broader impact of medication on an individual's overall mental health profile.

Second, there is a strong plausible mechanism linking ADHD medication initiation to each of these outcomes. ADHD pharmacotherapy is expected to reduce inattention and impulsivity, directly lowering the risk for accidental injuries. The effects on internalizing disorders are more complex; stimulant medications can sometimes exacerbate anxiety in

susceptible individuals, while non-stimulants like atomoxetine may have anxiolytic effects. Both medication classes can impact mood, making depression a critical outcome to monitor. Similarly, by treating core ADHD symptoms, medication may reduce the long-term risk of developing substance use disorders, making it a vital outcome to assess even in pediatric populations. Third, these outcomes occur with sufficient frequency in our population-based cohort to provide adequate statistical power for a within-individual analysis. In contrast, other valid but rarer comorbidities, such as psychosis or eating disorders, would result in very few events in the pre- and post-initiation periods for many individuals. Attempting to model such low-frequency events would lead to unstable estimates and an unacceptably low power to detect true associations, making the results unreliable. Our selection, therefore, prioritizes robust, interpretable findings for the most impactful clinical domains.

The 1-year pre-initiation period was chosen to establish a stable and representative baseline rate of events for each individual. A shorter period, such as 3 or 6 months, would be susceptible to random fluctuations, seasonality, or acute but transient life events, potentially providing a misleading baseline. A 1-year window smooths out this short-term variability and is long enough to capture events that may occur infrequently, providing a more robust estimate of an individual's typical risk before treatment. The 1-year post-initiation period was selected to allow sufficient time for the full effects of treatment to manifest and stabilize. This duration includes not only the immediate pharmacological effects but also the critical clinical processes of dose titration, stabilization on an effective dose, and the emergence of secondary benefits (e.g., improved functioning leading to reduced stress) or longer-term adverse effects. A shorter window would risk underestimating the true impact by focusing only on the acute phase of treatment.

Crucially, extending the follow-up period beyond one year (e.g., to two years) would introduce significant threats to the validity of the within-individual design. The core strength of this design is that individuals serve as their own controls, which assumes that confounders are stable over the observation window. Over longer periods, the risk of time-varying confounding increases; individuals undergo major developmental shifts, experience significant life events

(e.g., school transitions), and may start or stop other non-pharmacological interventions, making it impossible to attribute observed changes solely to ADHD medication. Furthermore, over a longer post-initiation period, treatment patterns become more complex, with higher rates of medication switching and discontinuation. This would contaminate the analysis, which is intended to assess the impact of initiating a specific treatment, not the mixed effects of a long and varied medication history.

Supplementary Table 1. Psychiatric diagnoses and negative outcomes by ICD-10 code

Diagnosis/Adverse Event Category	ICD-10 Code(s)	Notes/Specifications
Inpatient psychiatric hospitalizations	F00–F99	Defined by any primary or secondary discharge diagnosis.
Accidental injuries or accidents	S00–T78	Injury, poisoning, and certain other consequences of external causes (ED visits or hospitalizations).
External causes of morbidity	V00–X59	Used in relation to accidental injuries/accidents (ED visits or hospitalizations).
Alcohol-related disorders	F10	Identified from specialist care visits.
Drug-related disorders	F11–F16, F18–F19	Excluding alcohol and caffeine. Identified from specialist care visits.
Depressive disorders	F32–F33, F34.1	Includes dysthymia. Identified from specialist care visits.
Anxiety disorders	F40–F42	Includes phobic anxiety disorders and OCD. Identified from specialist care visits.
ADHD	F90	Attention Deficit Hyperactivity Disorder.
ASD	F84	Autism Spectrum Disorder.

ICD, International Classification of Diseases, 10th revision; ED, emergency department; OCD, obsessive-compulsive disorder.

Supplementary Table 2. Medication switching expressed as a rate per person-year on treatment, by age and diagnostic group.

**Subgroup**	**Switches**	**Person-years**	**Rate per person-year**	**Rate ratio (95% CI)**
Children, ADHD alone	66,443	100,416	0.66	1.00 (reference)
Children, ADHD+ASD	31,308	42,205	0.74	1.12 (1.07-1.18)
Adolescents, ADHD alone	37,015	111,067	0.33	1.00 (reference)
Adolescents, ADHD+ASD	8,518	24,954	0.34	1.02 (0.95-1.10)

Rate ratios are from quasi-Poisson regression with family-clustered standard errors; the reference group is ADHD alone within each age stratum. Time at risk runs from the first medication dispensation to the end of the three-year window. ADHD, attention-deficit/hyperactivity disorder; ASD, autism spectrum disorder; CI, confidence interval.

Supplementary Figure 1. Overview of the study design and the different index dates

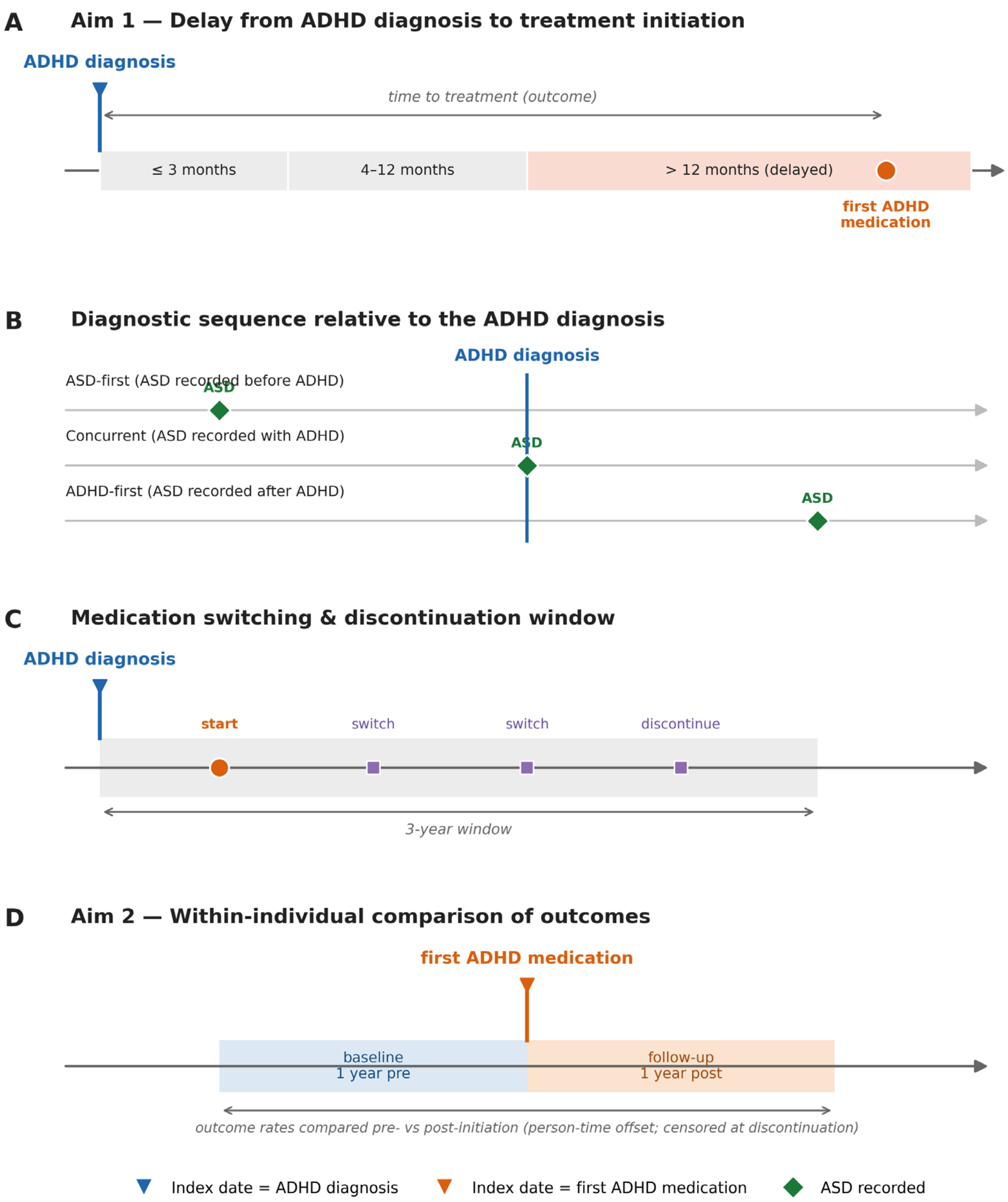

Supplementary Figure 1. Overview of the study design and the different index dates. (A) Aim 1: delay from the ADHD diagnosis (index date) to the first dispensed ADHD medication, categorised as ≤3, 4–12, or >12 months (delayed). (B) Classification of the diagnostic sequence according to whether ASD was recorded before (ASD-first), at the same time as (concurrent), or after (ADHD-first) the ADHD diagnosis. (C) Medication switches and discontinuations counted within the three-year window following the ADHD diagnosis. (D) Aim 2: within-individual comparison of outcome rates during the one year before versus the one year after the first ADHD medication (index date), with person-time modelled as an offset and follow-up censored at discontinuation. ADHD, attention-deficit/hyperactivity disorder; ASD, autism spectrum disorder.
